# Raman Spectroscopy in Cellular and Tissue Aging Research

**DOI:** 10.1111/acel.14494

**Published:** 2025-01-28

**Authors:** Jeong Hee Kim, Daejong Yang, Seungman Park

**Affiliations:** ^1^ Department of Mechanical Engineering Johns Hopkins University Baltimore Maryland USA; ^2^ Department of Mechanical and Automotive Engineering Kongju National University Cheonan Republic of Korea; ^3^ Department of Mechanical Engineering University of Nevada, Las Vegas Las Vegas Nevada USA; ^4^ Interdisciplinary Biomedical Engineering Program University of Nevada, Las Vegas Las Vegas Nevada USA

**Keywords:** aging, cell, Raman spectroscopy, senescence, tissue

## Abstract

The establishment of various molecular, physiological, and genetic markers for cellular senescence and aging‐associated conditions has progressed the aging study. To identify such markers, a combination of optical, proteomic‐, and sequencing‐based tools is primarily used, often accompanying extrinsic labels. Yet, the tools for clinical detection at the molecular, cellular, and tissue levels are still lacking which profoundly hinders advancements in the specific detection and timely prevention of aging‐related diseases and pathologies. Raman spectroscopy, with its capability for rapid, label‐free, and non‐invasive analysis of molecular compositions and alterations in aging cells and tissues, holds considerable promise for in vivo applications. In this review, we present recent advancements in the application of Raman spectroscopy to the study of aging in cells and tissues. We explore the use of Raman spectroscopy and related techniques for detecting cellular aging and senescence, focusing on the molecular alterations that accompany these processes. Subsequently, we provide a review of the application of Raman spectroscopy in identifying aging‐related changes in various molecules within tissues and organs.

## Graphical abstract

Aging is highly associated with changes in the functional properties and molecular compositions of cells and tissues. Raman spectroscopy is a non‐destructive chemical analysis technique that offers detailed insights into the molecular structure and compositions within cells and tissues. This review provides a comprehensive summary of findings from existing studies, offering a perspective on the application of Raman spectroscopy in aging research.

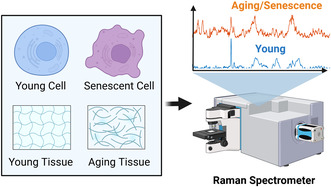

## 1 Introduction

Aging induces diverse functional properties, including molecular or biochemical, mechanical or viscoelastic, and structural or morphological properties, in organelles, cells, and tissues (Park 2022; Yang et al. 2023). Morphologically, an aging or senescent cell's nucleus and cell body become enlarged and irregular compared to the normal cell (Davies et al. 2022). An increase in F‐actin content and polymerization was notably observed in aging cells (Metzner et al. 2022). Mitochondria's phenotypes and activity were also changed in aging cells compared to normal cells (Miwa et al. 2022; Sun, Youle, and Finkel 2016). Senescent cells, a subset of aging cells, are known to secrete pathological molecules, senescence‐associated secretory phenotypes (SASPs) (Cuollo et al. 2020), and display increased lysosomal content (Tan and Finkel 2023) and senescence‐associated heterochromatin foci (SAHFs) (Aird and Zhang 2013). Mechanical or transport properties of aging or senescent cells and their nuclei were also found to be significantly altered (Bajpai, Li, and Chen 2021; Park and Kim 2022; Phillip et al. 2015).

At the tissue level, it has also been reported that tissue undergoes drastic changes in morphology and properties with age (Nemoto et al. 2012; Park et al. 2023, 2019). Studies have shown that the epidermis of chronologically aged skin layer becomes thinner than young skin, but that of photo‐aged skin becomes heterogeneously thickened in the epidermis (Costello et al. 2022). It was reported that collagen fibers tend to be more aligned in the aging skin tissue than in young, healthy skin tissue displaying randomly oriented networks (Kaur et al. 2019). Mechanical or viscoelastic properties of skin tissues were altered in the aging tissues (Krueger and Luebberding 2017; Pawlaczyk, Lelonkiewicz, and Wieczorowski 2013). In addition to the aforementioned features, many other proteins or molecules, such as collagen crosslink, collagen density/fragmentation, GAG/proteoglycan, matrix metalloproteinases (MMP), water content, and plasma viscosity, were found to be substantially changed (Freitas‐Rodríguez, Folgueras, and López‐Otín 2017; Park 2022).

Despite the diverse known hallmarks associated with the aging process, detecting cellular aging or senescence remains highly challenging due to the presence of heterogeneous phenotypes and the lack of highly specific and reliable biomarkers (Zimmermann et al. 2022). Another challenge arises from the variability in aging‐related features depending on the factor or stressor facilitating aging as well as cell type (Chen, Yang, and Jiang 2021; Dominic et al. 2020). For instance, cells undergoing stress‐induced aging, such as those exposed to disturbed flow or cancer therapy, exhibit distinct characteristics such as heightened levels of SASPs, increased reactive oxygen species (ROS), and greater telomere deoxyribonucleic acid (DNA) damage and dysfunctions in the Shelterin complex compared to cells undergoing time‐dependent aging (Dominic et al. 2020).

Recent advancements in Raman spectroscopy in the fields of biology and medicine offer promising avenues for detecting various aging‐related features and molecular changes within cells and tissues (Pezzotti 2021). Raman spectroscopy provides several advantages, including label‐free analysis, chemical specificity, non‐invasive measurement of cellular and tissue composition, structure, and dynamics, as well as high spatial resolution and in situ analysis (Watanabe, Sasaki, and Fujita 2022). However, despite these capabilities, the application of Raman spectroscopy remains underexplored in detecting cellular aging, senescence, and aging‐related molecular, structural, and mechanical changes (Varela‐Eirín and Demaria 2022).

In this review, we aim to explore the application of Raman spectroscopy to aging cells and tissues. First, we discuss the utilization of Raman spectroscopy and related techniques for the detection of cellular aging/senescence and related molecular alterations occurring during the aging/senescence process. Second, we provide a summary of the application of Raman spectroscopy in identifying diverse aging‐related changes within tissues and organs. Through this review, we aim to shed light on the promising role of Raman spectroscopy in advancing our understanding of aging and senescence of cells and tissues, potentially offering valuable insights for both basic research and clinical applications.

## 2 Applications of Raman Spectroscopy for Cellular Senescence and Aging Research

**FIGURE 1:**
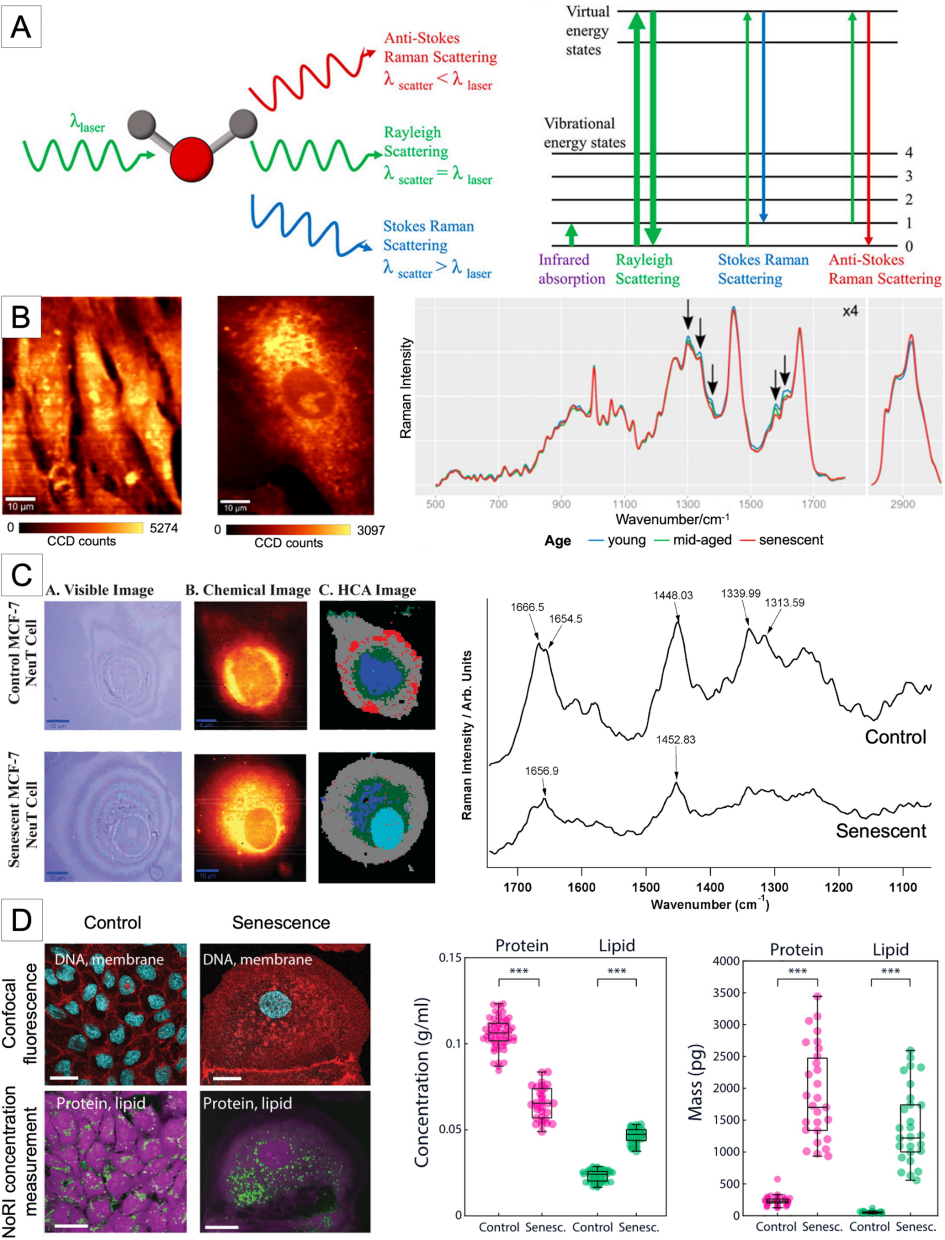
Application of Raman spectroscopy in cellular aging and senescence. (A) The principle of Raman scattering. As matter interacts with incident light (laser), light is either absorbed or scattered. The majority of the scattered light preserves the same frequency as the incident light (Rayleigh scattering), while a small fraction of light inelastically scatters (Raman scattering) with changes in net energy—either energy gain or loss—inducing an internal molecular vibration motion. Applications of Raman spectroscopy for studying (B) replicative senescence of primary human fibroblast cells, (C) oncogene‐induced senescence of epithelial breast cancer cells, and (D) therapy‐induced senescence of MDCK and A7 cells. (Reprinted with permission from (Liu et al. 2022), (Eberhardt, Beleites, et al. 2017), (Mariani et al. 2010), and (Oh et al. 2022)).

Over the past several decades, Raman spectroscopy has been extensively applied for studying various types of cells, owing to its molecular profiling capability that enabled us to infer biochemical makeups and molecular structures of cells (Figure 1A). In this section, we will focus on examples of how Raman spectroscopy has been applied to study cellular aging and senescence. Cellular senescence is defined as irreversible cell cycle arrest. Since its first demonstration in 1965 by Hayflick, the term, “cellular senescence”, has been commonly interchangeably used with “replicative senescence”, which, in fact, can be sub‐categorized as a type of cellular senescence (Hayflick 1965). The understanding of senescence has evolved and cellular senescence is found to be triggered by various factors such as oxidative stress, exposure to UV light, chemicals, and drugs. Thus, we focus on three main types of cellular senescence, categorized by its induction mechanisms: replicative senescence, oncogene‐activated senescence, and therapy‐induced senescence (Figure 1B–D). We categorize these studies based on three distinct Raman techniques: spontaneous Raman scattering, surface‐enhanced Raman scattering (SERS), and coherent Raman scattering (Table 1).

**TABLE 1:** Applications of Raman spectroscopy for studying cellular senescence and aging.

Cell line/type		Characterization method	Senescence/aging induction method	References
Fibroblasts	BJ, IMR‐90, MRC‐5, WI‐38	Spontaneous Raman	In vitro culture with population doublings from 28 to 72	Eberhardt, Beleites, et al. (2017) and Eberhardt et al. (2018)
	Human dermal fibroblasts	Spontaneous Raman	In vitro culture passages of 4–7 and 20	Eberhardt, Matthäus, et al. (2017)
	Hs 895.Sk	Spontaneous Raman	UV radiation (280–400 nm) for 50, 100, 200 min	Singh et al. (2017)
Cancer cells	MCF7	Spontaneous Raman	Facilitation of doxorubicin	Ghislanzoni et al. (2023)
	MCF7/NeuT	Spontaneous Raman	Facilitation of doxycycline hyclate	Mariani et al. (2010)
	U937	Spontaneous Raman	In vitro culture for 24 and 144 h	Fazio et al. (2016)
	HepG2	SRS, CARS	Facilitation of deferoxamine, doxorubicin, or radiotherapy	Bresci et al. (2023)
	MDCK, A7	SRS	Facilitation of doxorubicin	Oh et al. (2022)
Oocytes	CD‐1	Spontaneous Raman	Oocytes collected from young (4–8 weeks) vs. old (48–52 weeks) mice	Bogliolo et al. (2013)
Meschemal stem cells (MSCs)	Human umbilical cord MSCs	Spontaneous Raman	In vitro culture passages of 5, 10, 20, and 30	Bai et al. (2015)
Red blood cells (RBCs)	Human RBCs	Spontaneous Raman	0, 6, 13, and 22 days after blood sampling	Dinarelli et al. (2018) and Lenzi et al. (2021)

### 2.1 Detection of Senescent Cells Using Raman Spectroscopy

#### 2.1.1 Spontaneous Raman Scattering

Spontaneous Raman spectroscopy utilizes a laser emitting monochromatic light, typically ranging from the ultraviolet (UV) to near‐infrared (NIR) spectrum (Benevides, Overman, and Thomas 2005; Sato et al. 2024). When photons from the laser interact with molecules in the sample, they produce both elastically and inelastically scattered photons, termed Rayleigh scattering and Raman scattering, respectively. Raman spectroscopy quantitatively detects inelastically scattered photons while elastic ones are filtered out. The resulting Raman spectrum is highly specific to the sample, akin to the uniqueness of a human fingerprint, thereby referring to the technique as molecular fingerprinting. Raman spectroscopy allows for the detection and characterization of the various components within a heterogeneous sample, including biological cells, tissues, and organs. Particularly for the analysis of biological samples containing water molecules, Raman spectroscopy, compared to other vibrational spectroscopies, is favorable due to its little interference with water, facilitating measurement directly from biological samples without further preparation steps required. Typically, multiple spectra are gathered from a single sample to accommodate its heterogeneity. A significant advantage of Raman spectroscopy lies in its capability to identify multiple molecules contributing to a single spectroscopic signature rather than targeting specific biomarkers within the sample, enabling specific and sensitive molecular profiling of diverse molecules spontaneously.

##### 2.1.1.1 Replicative Senescence

Fibroblast cells have been one of the frequently investigated cell types with senescent features using Raman spectroscopy. Eberhardt and colleagues utilized four different strains of primary human fibroblast cells collected from the fetal foreskin, including BJ, IMR‐90, MRC‐5, and WI‐38 (Eberhardt, Beleites, et al. 2017). They separated cells into young, mid‐aged, and senescent groups based on their population doubling (PD) and observed notable changes in peaks attributable to proteins (1658 cm^−1^), lipids (1732, 2850, and 2930 cm^−1^), and nucleic acids (1580 cm^−1^) for all four strains despite slight variations presented in Raman spectra among different cell strains (Figure 1B). In addition, partial least squares‐linear discriminant analysis (PLS‐LDA) was used to objectively classify different age groups, resulting in a clear separation between young and senescent groups. However, some overlaps with mid‐age groups were presented for BJ and WI‐38 strains, which may be caused by the heterogeneity in cells transitioning into senescence. The prediction capability of the PLS‐LDA classifier with leave‐one‐PD‐out cross‐validation demonstrated up to 92% accuracy in predicting young and senescent groups, indicating the potential of Raman spectroscopy as a tool to identify cellular senescence. The group further explored fibroblasts cultured in three‐dimensional (3D) models in order to mimic in vivo conditions (Eberhardt, Matthäus, et al. 2017). Interestingly, compared to two‐dimensional (2D) culture conditions where no significant changes in Raman spectra between young (passages 4, 6 and 7) and senescent (passage 20) cells were observed, 3D cultures featured a clear distinction between the two groups, particularly in the 600–1260 cm^−1^, attributable to the changes in the proteins. From this study, they discovered significant differences in the biochemical behavior of senescent cells cultured in a 3D matrix compared to fibroblasts in a 2D condition, implying that in vitro studies would not sufficiently resemble the metabolic processes of cells in human skin. The PLS‐LDA model trained on spectra of cells (passages 4 and 20 only) cultured in 3D condition demonstrated 96% prediction accuracy when tested cells in 2D culture, while the reverse where the model trained with 2D and tested with 3D cultures failed to make an accurate prediction, indicating that cells cultured in 2D conditions lack metabolic properties of those in 3D models. One of the interesting findings from this study was the difference in morphological changes depending on cultivation conditions in that while they reported a noticeable increase in cell size of senescent cells in 2D, a well‐known senescent feature, they observed no changes in cell size in 3D cultures. Furthermore, they distinguished senescent cells from quiescent control cells, which can return to the cell cycle as opposed to senescent cells' permanent exit (Eberhardt et al. 2018). The PLS‐LDA analysis showed clear separation among proliferating, quiescent, and senescent cells, featuring differences in the Raman intensity ratios of proteins to lipids (1652 and 1454 cm^−1^), and nucleic acids (782 cm^−1^). They reported that compared to the other two cell states, senescent cells demonstrated the highest lipid and the lowest nucleic acid contents. Bai et al. (2015) examined mesenchymal stem cells isolated from human umbilical cord tissues (hUC‐MSCs), which were cultured for passages of 5, 10, 20, and 30 to monitor aging‐related changes. They identified the peak ratios of 1157 and 1174 cm^−1^ as potential indicators of cellular senescence, which showed a constant decline as the passage number increased, while the trend was not obvious for each peak alone. They also reported an expansion in full‐width half maximum (FWHM) at 934 cm^−1^. Taken together with the observation, the aging of hUC‐MSCs was found to be associated with protein cleavage and an increase of free amino acid residues. Raman spectra were collected from the cell center, presumably a signal predominantly arising from the nucleus.

##### 2.1.1.2 Oncogene‐Activated Senescence

Mariani et al. (2010) examined senescent epithelial breast cancer cells induced by oncogenic ErbB2 overexpression after doxycycline treatment using Raman spectral mapping. They observed typical morphological phenotypes as observed in other cellular senescence, including enlarged cells and granular cytoplasm. They focused on the nucleus region within Raman mapping of 10 individual cells and identified that the oncogenic cellular senescence, compared to the control breast carcinoma, featured changes in peaks at 1652 and 1666 cm^−1^ that are associated with *‐cis* and *‐trans* unsaturated fatty acid isomers present in cells with stable nuclear envelope architecture. While control cells showed two distinct peaks, senescent cells exhibited only a single peak of 1652 cm^−1^, indicating destabilized nuclear envelope organization, thereby leading to increased nuclear fluidity. In addition, they found that two peaks of 1313 and 1339 cm^−1^, present in control, disappeared in the senescent cell nucleus, indicating a decline in glycoproteins that are typically dispersed in the nuclear membrane (Figure 1C). They confirmed these observed Raman spectral changes using RT‐qPCR, which showed significantly downregulated expression of Nucleoporin 210 (NUP210) in senescent cells.

##### 2.1.1.3 Therapy‐Induced Senescence

Therapy‐induced senescent cells demonstrate certain morphological phenotypes distinct from those induced by other pathways, and for breast cancer cells, cell engulfment is one of the identified yet enigmatic features. An experimental study investigated by Ghislanzoni et al. (2023) focused on identifying biochemical features of senescent cells undergoing engulfment. They used human breast adenocarcinoma cells (MCF7) and treated them with doxorubicin to induce cellular senescence. Three different stages of the cell engulfment process were monitored: (1) fully engulfed, (2) degrading of the engulfed cell and partial formation of vacuole within the engulfing cell, and (3) complete vacuole formation within the engulfing cell after degradation of the engulfed cell. While they did not find a clear distinction among the three different engulfment stages, they could distinguish the Raman spectra of the engulfing cells from the vacuole. Furthermore, they reported that vacuoles formed after completing the degradation of engulfed cells are rich in lipids (1454 cm^−1^) with little protein contents (1007 and 1656 cm^−1^), distinct from both senescent cells and the surrounding medium. This study is intriguing as it investigates the phenotypic features of therapy‐induced senescence in relation to their biomolecular variations. Findings in this study can be used to offer insights into senescent cells becoming therapy‐resistant and supplying nutrients to senescent cells.

#### 2.1.2 Surface‐Enhanced Raman Scattering (SERS)

Surface‐enhanced Raman scattering (SERS) exploits plasmonic effects arising from the light interaction of molecules with special substrates, typically containing nano‐sized metal structures (Tanwar et al. 2022). The Raman signal of molecules in close proximity to the substrate undergoes an field concentration, which in turn contributes to the substantial enhancement of the signal (Schatz, Young, and Duyne 2006).

##### 2.1.2.1 Therapy‐Induced Senescence

Senescent cells are known to secrete SASP factors, including extracellular vesicles (EVs), inflammatory cytokines, or molecules. Lee and colleagues investigated EVs, particularly those of small size (30 nm–200 nm), which are mainly exosomes derived from ionizing irradiation‐induced senescent cells (Lee et al. 2022). They tested human lung fibroblasts (IMR90) to establish senescent and quiescent (control) types to secrete EVs, of which small EVs were isolated and prepared on silver nanoforest substrate for SERS measurement. Their work demonstrated that small EVs secreted by senescent cells featured distinct spectral profiles from quiescent control cells with clear separation in principal component analysis (PCA) scores in the first three principal components (PCs), which represented 84.4% of the variability. The first 10 PCs of PCA analysis were subjected to hierarchical clustering analysis, which identified among observed Raman peaks, those attributable to amino acids (872, 1442, 1322, 1522, and 1609 cm^−1^) substantially contributed to the distinction between spectra of senescent and quiescent EVs. The observed peaks were further analyzed by identifying peaks with respect to the net charge of contributing amino acids, namely arginine, histidine, and lysine for the positive net charge and aspartic acid and glutamic acid for the negative net charge, showing that the intensity summation of peaks representative of both positively and negatively charged amino acids in senescent small EVs were greater than those of quiescent small EVs. Furthermore, the intensity ratio of positively charged to negatively charged amino acid peaks for the senescent was visibly greater than the control, concluding that changes in the Raman profiles are associated with the change in surface charge of small EVs.

#### 2.1.3 Coherent Raman Scattering

Non‐linear coherent Raman spectroscopy is a popular variant for studying biological specimens due to its chemical specificity (Krafft et al. 2017; Parodi et al. 2020; Zhang et al. 2014). This technique devises Stokes laser in addition to a single pump laser for illumination, producing enhanced Raman signal due to the vibrational coherence of molecules.

##### 2.1.3.1 Therapy‐Induced Senescence

Oh and colleagues have developed a new approach for quantitatively profiling the physical properties of cells, including protein and lipid concentration, based on a stimulated Raman scattering (SRS) technique by computationally processing artifacts induced by the light scattering effect (Oh et al. 2022). They quantified changes in cytoplasmic concentration of MDCK and A7 cells that were induced to senesce by doxorubicin treatment. In addition, a decrease in cytoplasmic dry mass and protein concentration for senescent cells was observed, compared to proliferating control. Lipid accumulation of senescent cells was also quantified, demonstrating a statistically significant increase in total lipid concentration (0.023–0.047 g/mL), total lipid mass (50.5–1377.3 pg), and lipid‐to‐protein proportion (0.53–1.44) for MDCK cells (Figure 1D). On the other hand, A7 cells showed even protein and lipid accumulation for the senescent phenotype, indicating cell type‐dependent heterogeneity in cellular senescence and its metabolic reprogramming.

A combination of non‐linear Raman techniques, including SRS and coherent anti‐Stokes Raman scattering (CARS), was used to characterize therapy‐induced senescence (Bresci et al. 2023). Bresci and colleagues employed deferoxamine to human liver cancer cells (HepG2) to establish therapy‐induced senescence. Co‐registered images of each modality localized lipid vesicles, cellular proteins, and nuclei, characterized by 2850, 2930, and 2970 cm^−1^, respectively. They could monitor temporal changes in these subcellular structures spanning from 0 h up to 7 days of senescence induction, which revealed lipid droplet accumulation and mitochondrial network rearrangement in senescent cells. On the other hand, proliferating cancer cells maintained constant over the 7 days of observation. Based on the chemical maps captured by multi‐modal nonlinear Raman techniques, the authors quantified changes in lipid droplets, displaying a significant increase in lipid droplet occupation within senescent cells. They further validated this observation with radiotherapy‐induced senescent HepG2 cells.

### 2.2 Molecular and Protein Changes in Cells During Aging

As cells age, they undergo significant molecular or protein changes. Raman spectroscopy enables the obtaining of multiplexed biochemical or molecular information from various molecular species, such as proteins, lipids, DNA, and mineral deposits (Table 2). Bogliolo et al. (2013) focused on the influence of age‐related oxidative damage on the oocytes of CD‐1 mice. They employed oocytes collected from young mice (4–8 weeks) and old mice (48–52 weeks) and separated them into 4 groups, including (1) young oocytes, (2) in vitro aged oocytes (10 h culture), (3) oxidative‐stressed oocytes (hydrogen peroxide treated) from young mice, and (4) old oocytes from reproductively old mice. They identified variations in Raman peaks indicative of proteins and lipids between young and aged oocytes. PCA of Raman spectra clearly separated young oocytes from aged oocytes with loading plots featuring peaks attributable to lipids (1211, 1345, 1450, and 1659 cm^−1^) and proteins (1035, 1132, 1450, and 1659 cm^−1^). However, no distinct separation among different aged groups was observed, indicating that developmental abnormalities due to aging are associated with chemical modification induced by oxidative stress.

**TABLE 2:** Raman band assignments associated with cellular senescence and aging.

Raman shift (cm^−1^)	Peak assignment			References
	Proteins	Lipids	Nucleic acids	
835	Tyrosine			Singh et al. (2017)
934	C–C backbone stretching vibrations			Bai et al. (2015)
1000, 1003, 1035	Phenylalanine			Bogliolo et al. (2013), Dinarelli et al. (2018), and Singh et al. (2017)
1081			Nucleic acids	Hsiao et al. (2021)
1120	Proteins			Hsiao et al. (2021)
1124	Glucose			Hsiao et al. (2021)
1132, 1157	C−C, C−N stretching vibrations			Bai et al. (2015) and Bogliolo et al. (2013)
1174	C−H bending vibrations in tyrosine and phenylalanine			Bai et al. (2015)
1211		OH bending C−N stretching		Bogliolo et al. (2013)
1313, 1339	Glycoproteins			Mariani et al. (2010)
1345		CH_3_ deformation		Bogliolo et al. (2013)
1430, 1440, 1450	CH_2_ bending CH_3_ deformation	CH_2_ bending		Bogliolo et al. (2013), Lenzi et al. (2021), and Singh et al. (2017)
1584			−N−H bending vibration of guanine or adenine residues	Singh et al. (2017)
1640	Amide I			Lenzi et al. (2021)
1652, 1666	Amide I	Unsaturated membrane lipids of *‐cis* and *‐trans* isomers		Eberhardt et al. (2018) and Mariani et al. (2010)
1658, 1659, 1660	Amide I	C=C stretching		Bogliolo et al. (2013), Eberhardt, Beleites, et al. (2017), and Singh et al. (2017)
2850, 2853		CH_2_ stretching vibrations Methylene groups		Bresci et al. (2023) and Oh et al. (2022)
2935	Methyl groups			Oh et al. (2022)
2970			Deoxyribose CH− stretching vibrations	Bresci et al. (2023)

Fazio and coworkers investigated a leukemia model, established by a human U937 cell line, cultured in vitro for 24 and 144 h to observe molecular changes induced by culture time (Fazio et al. 2016). They reported that the aged culture, compared to the fresh culture, showed increased cell‐to‐cell variability, featured by variations in Raman intensity of protein (1315–1330, 1450–1458, 1658–1671 cm^−1^), lipid (1263–1264, 1300–1303, 1439–1442, 1650–1657 cm^−1^), and nucleic acid bands (775–803, 811–815, 1093–1097, 1370–1374, 1576–1578 cm^−1^). Also, these changes were demonstrated in aged cultures with heterogeneous cell morphology and nucleus structure with round shapes and vesicles budding from the nucleus, respectively.

Red blood cells (RBCs) isolated from human blood samples have been used for the application of Raman spectroscopy (Dinarelli et al. 2018; Lenzi et al. 2021). Results revealed the contributions of proteins and membrane lipids during the aging process from 0 to 22 days after blood sampling, synchronized with a morphological aging pattern of RBCs. The morphology of RBCs, initially biconcave (> 80% of cells until day 2), progressively shifts into crenated, spiculed, and flat/spherocytic (> 70% after day 22) shapes as they age. Raman spectra of cells with representative morphological shapes at days 0, 6, 13, and 22 showed a clear difference between young (days 0 and 6) and old (days 13 and 22) cells. An apparent intensity decrease between 900 and 1300 cm^−1^ was observed, indicating changes in mechanical properties and fluidity of the cell membrane mainly due to the disintegrity of lipid and protein conformation. Thus, cell morphological and biochemical changes profiled by Raman spectroscopy were correlated, which demonstrated that the changes in the two are associated with one another. The authors further performed PCA analysis on RBC spectra of all shapes at each time point, yielding a distinct separation between young and old cells across PC1 domain.

UV radiation‐induced damage in skin fibroblasts was studied by using Raman spectroscopy (Singh et al. 2017). Results showed structural changes in lipids and proteins, featured by changes in amide I (1660 cm^−1^), amide III, tyrosine (835 cm^−1^), and 1440 cm^−1^ bands. They also found an increase in peaks at 1000 and 1584 cm^−1^ with increasing exposure time, which are attributed to phenylalanine and guanine or adenine within DNA, respectively, indicating that exposure to UV radiation may activate cells' apoptotic pathways. They further performed PCA, which showed a clear separation between UV irradiated cells and the control group with PC loadings exhibiting the features mentioned above. PCA results thus demonstrated that molecular changes in fibroblasts due to UV radiation can be objectively identified. Moreover, biochemical changes observed from Raman spectra with changes in 3D morphology collected by quantitative phase imaging were correlated. Quantitative phase images unlocked biophysical properties, including cell dry mass and density, which exhibited a statistically significant reduction in both measures as the exposure continued.

Changes in telomere length are one of the well‐known features associated with cellular senescence and aging that constantly decreases over time with cell divisions (Bernadotte, Mikhelson, and Spivak 2016; Lai, Wright, and Shay 2018). Telomeres play a critical role in protecting the chromosome ends, thereby maintaining genome stability, and the progressive shortening triggers DNA damage response, leading to replicative senescence (Blasco 2007; Lai, Wright, and Shay 2018). Using SERS, telomere length was optically measured (Zong et al. 2014). They employed two SERS probes targeting genome DNA extracted from cells with one specific to telomere and the other specific to centromere. SERS signal was internally calibrated with centromere‐specific probes, and telomere‐specific probes yielded a stronger signal with longer telomere length, thereby enabling length measurement. They quantified telomere length by calculating the ratio of peaks characteristic to telomeres and centromeres, 1648 and 1622 cm^−1^, respectively, demonstrating a constant decrease with increasing population doubling levels of MRC‐5 fibroblasts. While this study is different from other studies discussed in this review that optically measured telomere length rather than probing molecular changes due to cellular senescence, it holds significance that suggests a new avenue of applying Raman spectroscopy for assessing cellular senescence.

## 3 Applications of Raman Spectroscopy for Aging Research in Tissues

Aging analysis of tissues and organs using Raman spectroscopy is more challenging compared to cellular analysis due to tissue complexity, heterogeneity, depth of penetration, and interference from surrounding components (Pereira et al. 2021). Nevertheless, numerous studies have been conducted to apply Raman spectroscopy to aging tissues and organs in order to evaluate their aging state and to observe molecular changes occurring during the aging process (Ager et al. 2005; Gamsjaeger et al. 2010; Miyamori et al. 2021; Nguyen et al. 2013; Pereira et al. 2021; Van Gulick et al. 2019). Research has recently become active, with studies reported on skin, bone, tendon, and eye tissue (Table 3). Compared to cellular‐level studies, studies of tissues and organs are mostly conducted using spontaneous Raman scattering, with very few uses of surface enhancement such as SERS and surface‐enhanced coherent anti‐stokes Raman scattering (SECARS). Tissues and organs need to be measured over a wider range than cells and it requires techniques to uniformly embed metal particles for surface enhancement. In this section, we review studies of aging at the tissue and organ level using Raman spectroscopy.

**TABLE 3:** Raman band assignments associated with tissue aging.

Tissue/organ	Peak assignment	Raman shift (cm^−1^)	References
Skin (dermis)	Amide I band	1605, 1615, 1635, 1658, 1668, 1688	Nguyen et al. (2013)
Skin (stratum corneum, epidermis)	Carboxymethyl‐lysine	454, 548, 752, 852, 926, 1078, 1156, 1360, 1418, 1460, 1522	Pereira et al. (2021)
Skin (dermis)	Glucosepane	438, 492, 580, 600, 840, 902, 1048, 1064, 1102, 1276, 1480, 1658	Pereira et al. (2021)
Skin (dermis)	Pentosidine	834, 868, 916, 1264, 1344, 1470, 1506, 1616, 1636	Pereira et al. (2021)
Skin (sub‐umbilical, abdominal)	Amide I	1652 (v(C=O), α‐helix), 1681 (v(C=O), disordered structure)	Miyamori et al. (2021)
Bone (cortical)	Amide I band	1610, 1655	Ager et al. (2005)
Bone (femurs, mouse)	Carbonate group	1070 (ν_1_)	Gamsjaeger et al. (2010)
Bone (femurs, mouse)	Phosphate group	428 (ν2), 592 (ν4), 960 (ν_1_)	Gamsjaeger et al. (2010)
Bone (femurs, mouse)	Amide I band	1670	Gamsjaeger et al. (2010)
Eye (Bruch's membrane)	AGEs	880, 980, 1080–1090	Glenn et al. (2007)
Tendon (tail, rat)	Proline	854 (anisotropic polarization)	Van Gulick et al. (2019)
Tendon (tail, rat)	Amide III band	1243 (anisotropic polarization)	Van Gulick et al. (2019)
Tendon (tail, rat)	Amide I band	1668 (anisotropic polarization)	Van Gulick et al. (2019)

### 3.1 Detection of Aging Tissues Using Raman Spectroscopy

**FIGURE 2:**
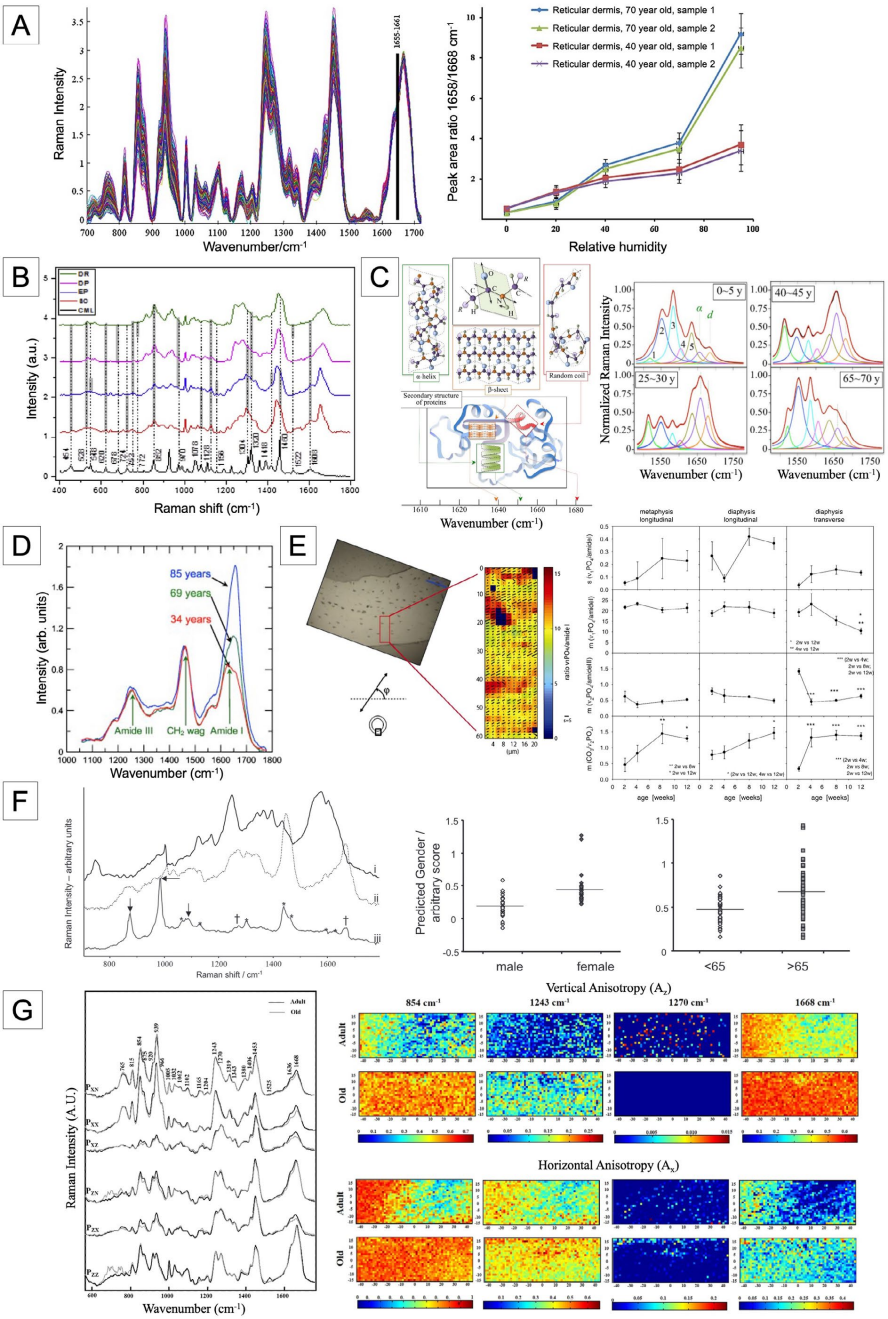
Raman signal‐based aging analysis at the tissue level. (A) (left) Raman spectrum of the dermal skin with age and (right) differences in Raman peak area ratio with aging. (B) Raman spectrum of skin layers (reticular dermis (DR), dermal papillae (DP), epidermal (EP), and stratum corneum (SC)) and peak marking of carboxymethyl‐lysine (CML) for the photo‐exposed group. (C) (left) Raman spectrum analysis based on protein folding mechanisms and (right) differences in Raman spectrum by age. (D) Differences in the Raman spectrum of bones with aging. (E) (left) Collagen fibril orientation map obtained by analyzing polarized Raman signal and (right) changes in molecular organization and orientation with age. (F) Raman analysis of heme, collagen, and AGEs in Bruch's membrane and correlation with gender and age. (G) Polarized Raman spectroscopy spectrum of adult and old rats and their mapping results. (Reprinted with permission from (Nguyen et al. 2013), (Pereira et al. 2021), (Miyamori et al. 2021), (Ager et al. 2005), (Gamsjaeger et al. 2010), (Glenn et al. 2007) and (Van Gulick et al. 2019)).

Skin tissue has been frequently utilized for applications in Raman spectroscopy. Different layers of the skin, such as stratum corneum (Boireau‐Adamezyk, Baillet‐Guffroy, and Stamatas 2014; Choe et al. 2018; Egawa and Tagami 2007; Pereira et al. 2021), epidermis (Pereira et al. 2021), and dermis (Nguyen et al. 2013; Téllez S et al. 2015) have been tested and analyzed based chronological age and after UV exposure. Although, oftentimes, studies have shown inconsistent or contradictory results between studies due to insufficient sampling numbers and Raman's low penetration depth, the majority of studies have recognized the amide I band (Nguyen et al. 2013) (Figure 2A), carboxymethyl‐lysine (CML), glucosepane (GLU), and pentosidine (PEN) (Téllez S et al. 2015) as key markers of aging. The amide I band is considered the most common factor in determining skin aging. In the stratum corneum, CML can be used to evaluate aging, and, in the epidermis and dermis (Figure 2B), GLU and PEN can be used to indicate aging. Aging can also be estimated by measuring the protein‐folding intensity ratio of skin. Miyamori and colleagues calculated the ratio of v(C=O) in the disordered structure (1681 cm^−1^) and in the α‐helix (1652 cm^−1^) of Amide I (Figure 2C) and created an expression that converts to age (Miyamori et al. 2021). The protein‐folding intensity increased with increasing age, and the proposed formula showed a coefficient of determination of 0.743. Green, Ellis, and Winlove (2008) showed that UV‐induced aging results in specific alterations within the dermis, detectable through Raman spectroscopy at peaks 1246 and 1274 cm^−1^.

Raman spectroscopy has also been applied to evaluate bone aging. Ager et al. (2005) measured Raman spectra of human cortical bone from donors aged 34–99 years. They focused on amide I and found that the intensity of the 1610 cm^−1^ pick decreased and that of 1655 cm^−1^ pick increased with age, shifting the overall peak of amide I in the direction of a larger wavenumber (Figure 2D). Gamsjaeger et al. analyzed carbonate (${\mathrm{CO}}_{3}^{2-}$) and phosphate (${\mathrm{PO}}_{4}^{3-}$) using Raman spectroscopy (Figure 2E). They measured Raman spectra of the femurs of 2, 4, 8, and 12‐week‐old mice and quantified the intensity ratio of ${\mathrm{CO}}_{3}^{2-}$ , ${\mathrm{PO}}_{4}^{3-}$ , and amide I (Gamsjaeger et al. 2010). The ratio of CO_3_ to ν_2_PO_4_ was found to increase with age. This may imply that the amount of B‐type carbonate increases relative to phosphate during aging, which can be observed using Raman spectroscopy. In addition, by analyzing the ratio of ν_1_PO_4_ to amide I, it was suggested that the alignment of collagen fibrils increases with age.

For the ocular tissues, Glenn and colleagues showed that the accumulation of advanced glycation end products (AGEs) in Bruch's membranes is associated with aging (Glenn et al. 2007) (Figure 2F). Beattie et al. (2011) analyzed 67 eyes of aged 33–92 to correlate AGEs in sclera as a surrogate of Bruch’s membrane with aging. Experimental findings exhibited that the correlation with chronological age is lower for sclera (*R* ^2^ = 0.717) than for Bruch's membrane (*R* ^2^ = 0.824), indicating sclera can serve as a reliable marker for profiling AGE accumulation in Bruch’s membrane.

Van Gulick et al. (2019) suggested a novel method to measure the aging of tendons. They applied polarized Raman spectroscopy (PRS), which not only provides the chemical composition of the tendon but also the structural organization, using the tails of 2 and 24‐month‐old Wistar rats. The amide I (1668 cm^−1^), amide III (1243 cm^−1^), and proline (854 cm^−1^) related bands were highly sensitive to polarization and strongly associated with aging (Figure 2G). The degree of anisotropy of the Raman bands increases with age, indicating that in older tendons, the collagen fibers are more aligned with the fascial backbone axis, resulting in a higher degree of straightness of the collagen fibers.

### 3.2 Molecular Changes in Tissues During Aging

To date, Raman spectra and imaging have been extensively employed to investigate aging‐related changes in various molecules or proteins within tissues, including AGEs/ALEs (Advanced lipoxidation end‐products), water, lipids, and collagen. AGEs arise from the non‐enzymatic glycation of proteins and lipids within tissues and cells (Gkogkolou and Böhm 2012; Rungratanawanich et al. 2021). AGEs have the propensity to accumulate on nucleotides, lipids, and proteins throughout various tissues and cells as an individual ages, thereby altering the biophysical and biochemical properties of tissues or organs (Avery and Bailey 2006; DeGroot et al. 2001) and resulting in age‐related diseases and complications (Chaudhuri et al. 2018). Confocal Raman spectroscopy, a Raman technique combined with a confocal optical microscope, has been used to localize and quantify AGEs during aging in different tissues such as a Bruch's membrane from post‐mortem eyes (Glenn et al. 2007; Wang et al. 2023), human dermis tissue (Téllez and Claudio 2017), and in vitro collagen model (Alsamad et al. 2021). ALEs are intricate compounds resulting from the modification of lysine (Lys), arginine (Arg), cysteine, and histidine (His) residues in proteins, both exogenously and endogenously. Comparable to AGEs, aging prompts heightened accumulation of ALEs through lipid peroxidation reactions (Moldogazieva et al. 2019). Furthermore, both ALEs and AGEs as modified proteins are recognized for their pathogenetic role in the onset and advancement of aging‐related diseases, including diabetes, atherosclerosis, and neurodegenerative disorders (Mol et al. 2019; Vistoli et al. 2013). The Raman spectral bands used for detecting AGEs/ALEs vary depending on the specific type, including, methyl glyoxal (MGO) (609, 685, 809, and 1734 cm^−1^), pentosidine (801 cm^−1^), glyoxal (GO), (820, 1038, and 1276 cm^−1^), carboxymethyl lysine (CML) (930 and 1390 cm^−1^), carboxyethyl lysine (CEL) (1360 and 1405 cm^−1^), methylglyoxal‐lysine dimer (MOLD) (1400 cm^−1^), glyoxal‐lysine dimer (GOLD) (1420 and 1445 cm^−1^), Acrolein (1620 and 1655 cm^−1^), and others (Beattie, McGarvey, and Stitt 2013).

Water content has been quantified through Raman fingerprints during the aging process. Studies have demonstrated an increased water loss in transdermal skin tissue with age, accompanied by a decreased lipid/protein ratio (Egawa and Tagami 2007). However, another study displayed the opposite trend, revealing increased water content in the dermal tissue of aged women compared to healthy young women (Téllez S et al. 2015). Téllez‐Soto and colleagues measured the water content in collagen at the dermis using Raman signals. They analyzed the ν(OH) attached to water in the band between 3150 and 3700 cm^−1^ (Téllez‐Soto et al. 2021). The quantification of alterations in lipid composition due to aging was conducted using Raman microspectroscopy in murine perivascular adipose tissue (Czamara et al. 2018). The levels of carotenoid expression or macular pigment optical density were assessed using resonance Raman spectroscopy to explore the effects associated with aging (Bernstein et al. 2002; Obana et al. 2014).

Collagen is a major component of various tissues, including skin, heart, and cartilage (Reiser, McCormick, and Rucker 1992). Raman spectroscopy has been extensively applied to analyze conformational and structural changes in collagen molecules. This technique is particularly sensitive to two distinct secondary structures, contributing significantly to the stability and integrity of collagen fibrils, within the collagen macromolecule: the α‐helix and the β‐sheet (McColl et al. 2003). The frequencies of the Amide I and Amide III bands are particularly useful in detecting differences in secondary structures through Raman fingerprinting (Kim et al. 2023). This region, along with the lipid and proline/hydroxyproline regions, are the three main areas frequently involved in conformational changes of the collagen structure. Early findings demonstrated that 19% of the polypeptide chain adopts a β‐sheet conformation, while 18% adopts an α‐helix structure, displaying Amide I bands at 1672 and 1650 cm^−1^, respectively (Chou and Fasman 1974; Copeland and Spiro 1986; McColl et al. 2003). Gullekson and colleagues conducted the first characterization of collagen I fibrils from rat‐tail collagen using SERS (Gullekson et al. 2011). silver (Ag) and gold (Au) nanoparticles were functionalized onto collagen fibers to amplify the Raman signal of the samples. The results revealed that Raman spectra exhibited high sensitivity to the exposure of aromatic side chains on the surface of the fibrils. Specifically, residues within the helical structure of collagen were characterized by peaks at 1000 cm^−1^ for phenylalanine and 1605 cm^−1^ for both phenylalanine and tyrosine. In addition, the Amide I and Amide III bands were observed at 1655 and 1268 cm^−1^, respectively. Previous studies suggested that the ratios within the Amide I and Amide III bands can serve as diagnostic indicators of age‐related alterations in various collagen‐rich tissues, by quantifying the presence of mature crosslinks and assessing the disordered secondary structure of collagen (Dehring et al. 2006; Paschalis et al. 2001). To investigate age‐related alterations in the molecular composition of tendons, Yin and colleagues conducted a comprehensive stepwise analysis incorporating both fluorescence and Raman spectra, which enables a more nuanced understanding of the chemical distinctions between young and aged tendons (Yin et al. 2020). The results showed that the young group exhibited stronger Raman intensities at various peaks compared to the old group in both tendons, including wavenumbers between 800 and 1000 cm^−1^, which are typically associated with the collagen backbone. Van Gulick investigated age‐related modifications in the molecular organization of type I collagen in rat tail tendons using polarized Raman spectroscopy (Van Gulick et al. 2019). The Amide I and Amide III bands, as well as those associated with proline and hydroxyproline, were found to be highly sensitive to polarization and aging. Depolarization and anisotropic ratios were employed to elucidate changes in the orientation of collagen fibers with age. The degree of anisotropy of Raman bands increased from adult to old collagen, suggesting a higher alignment of collagen fibers to the fascicle backbone axis in older tendons, resulting in greater straightness of collagen fibers. Villaret and colleagues assessed the aging process by analyzing collagen from the papillary dermis, focusing on the following Raman peaks: 857, 922, 938, 1245, 1271, and 1451 cm^−1^ (Beattie, McGarvey, and Stitt 2013).

Compared to the aforementioned molecules and proteins, only a limited number of studies have explored elastin, proteoglycans, and other macromolecules in tissues using Raman spectroscopy to observe aging‐related molecular and structural changes. Further research is needed to elucidate these aspects more comprehensively.

## 4 Discussion

Label‐free, non‐invasive Raman spectroscopy has been widely used in the field of aging research (Liendl, Grillari, and Schosserer 2020). However, several factors and challenges to overcome should be taken into account in further studies. First of all, careful consideration should be given to accurately defining and characterizing senescent cells and aging cells before employing Raman spectroscopy, as they are often mistakenly used interchangeably (Ogrodnik 2021). Senescent cells are defined as cells that have stopped dividing and are in a state of irreversible growth arrest in response to cellular damage or stress. Aging cells are referred to as cells that are undergoing the aging process, which involves a gradual decline in cellular function over time. Senescent cells are considered a subset of aging cells, though the inverse relationship does not necessarily apply. It has been known that senescent cells can contribute to aging and age‐related diseases through the secretion of inflammatory molecules or cytokines, known as the SASPs, whereas not all aging cells necessarily produce them (Guo et al. 2022).

It is important to note that while Raman spectroscopy is valuable for identifying molecular changes or signatures associated with cellular senescence and aging, it does not directly detect senescent or aging cells. Furthermore, the characteristics of senescent cells are highly tissue‐specific, necessitating validation across various tissue types. To address this, spectral data obtained through Raman spectroscopy must be validated and correlated with established global molecular markers of senescence or aging, such as SA‐β‐gal or p16^INK4a^ (Cai et al. 2020; Safwan‐Zaiter, Wagner, and Wagner 2022). This validation step is crucial for enhancing the reliability of Raman spectroscopy as a tool for studying senescence and aging, ensuring its robustness, and providing a solid framework for its application in both experimental and clinical settings.

It is well‐established that cellular senescence and aging can be triggered by various aging‐causing factors or stressors, such as anti‐cancer drugs, UV light, disturbed flow, and more (Park et al. 2024). However, a prevailing issue in current research is the tendency for many studies to overgeneralize their results without distinguishing between stressor or aging types. Recent studies have unveiled substantial variances in cell cycle arrest, telomere shortening, DNA damage, shelterin complex dysfunction, and expression of SASPs between natural aging and stress‐induced aging. Hence, it is imperative to delineate aging‐related characteristics based on the specific stressor or type of aging.

Analyzing aging in tissues and organs using Raman spectroscopy presents a more challenging context than in cells. As demonstrated in the study by Pereira et al. (2021), different layers of the skin—the stratum corneum, epidermis, and dermis—exhibit distinct trends. This is due to the complexity of organs and tissues, and it is required to conduct research on each specific tissue and organ. However, this complexity can also be advantageous, as it provides both structural and material information. Notable examples include the work of Van Gulick and colleagues, as well as that of Gamsjaeger and colleagues. Van Gulick et al. (2019) utilized PRS to analyze the polarization of collagen fibers in rat tendons to determine their aging. Gamsjaeger et al. (2010) measured Raman spectra of mouse bone by rotating the laser's polarization direction, allowing them to analyze the composition and orientation of the analytes with age. Gender‐induced differences need to be considered as well. Although there has been little research on gender‐specific differences in aging or senescence using Raman (Glenn et al. 2007; Nieuwoudt et al. 2020), gender is an important factor to consider. The difference in bone components is one of the most significant examples. Nieuwoudt et al. analyzed the matrix ratios (MMR) and ${\mathrm{CO}}_{3}^{2-}$/${\mathrm{PO}}_{4}^{3-}$ ratios, and relative to lipid/collagen –CH_2_ deformation modes at 1450 cm^−1^ of cortical bone. While there were no significant differences between bone samples from males and females under age 62, significant differences in bone composition were observed between males and females over age 70, as well as between “old (≥ 70)” and “young (≤ 62)” groups (Nieuwoudt et al. 2020).

The utilization of the Raman spectroscopic technique is quite challenging within clinical practice, requiring complicated instrument setup, a long acquisition time for quality spectrum, and expertise in handling Raman spectral data (Elumalai, Managó, and De Luca 2020). Consequently, a pressing demand exists to develop an innovative automatic spectrometer enhancing efficacy or streamlining current instrumentation and the pipeline for data processing and analysis, prioritizing speed, as Raman spectroscopy presents challenges for facilitating wider adoption to users including biologists and clinicians without extensive training in biophotonics, bio‐optics, and chemometrics (Liendl, Grillari, and Schosserer 2020). To reduce acquisition time and enhance detection sensitivity, signal enhancement methods such as SERS and SECARS can be employed. However, when measuring large and thick areas of tissues and organs, it is essential to develop techniques that uniformly embed the metal nanoparticles to achieve the desired enhancement. Also, significant effort in streamlining Raman data processing and analysis has been demonstrated recently, combined with machine learning and deep learning models (Fang et al. 2024; Qi et al. 2023).

Another significant challenge arises from the lengthy acquisition time required for imaging. While Raman imaging offers molecular distribution mapping in addition to the characterization of biological specimens up to diffraction‐limited spatial resolution, making it excellent for monitoring cellular dynamics, its practice is restricted largely due to extensive measurement time. Since biological cells and tissues are weak Raman scatterers and Raman scattering is inherently a low‐probability process, achieving high signal‐to‐noise ratio (SNR) spectra necessitates prolonged measurement times. Consequently, Raman data acquisition, especially for high spatial resolution imaging, can take several hours to days. One study covered in this review, in fact, reported that experiments were adjusted from 11‐h long cell imaging to 4‐min long line scan in order to accommodate large sample size without sample degradation (Bogliolo et al. 2013). Increased acquisition time constraints sample conditions from using fresh, live cells to fixed or dried samples to curb sample‐to‐sample variability that may arise from different measurement time points. Most studies that performed Raman imaging utilized fixed cells (Czamara et al. 2017; Eberhardt, Matthäus, et al. 2017; Eberhardt et al. 2018; Fazio et al. 2016; Mariani et al. 2010). Such limitation hinders us monitoring from cellular dynamics and short‐to‐long‐term metabolic, phenotypic, and structural changes relevant to cellular senescence. Techniques including SERS and nonlinear Raman can alleviate this issue by producing highly sensitive signals by exploiting metallic structures or substrates and additional high‐power laser sources. Moreover, Raman spectroscopy exhibits high sensitivity to fluorescence interference originating from endogenous fluorophores present in cells or exogenous fluorophores utilized for labeling molecules or proteins for immunofluorescence. These fluorescence signals can interfere with Raman signals, thereby diminishing the technique's sensitivity and specificity. Time‐gated Raman spectroscopy can effectively address fluorescence interference, which can be potentially applied to cellular senescence and aging research in the future.

Additionally, the use of diverse protocols for sample preparation adds complexity in interpreting the Raman signal and making a cohesive decision based on the resulting Raman spectra. Various factors, including sample substrate, embedded condition, and preparation protocols, contribute to alternation in cells and tissues as well as background influence in Raman spectra. In order to minimize potential interference with the background signal, biological samples are often prepared on quartz or CaF_2_ slides, as these materials have a silent signal in the biological fingerprint region. However, protocols for sample preparation are diverse, and different protocols are practiced for cells and tissues. For Raman measurement, cells are typically prepared in live, fixed, or dried conditions. For measuring live cells, Raman spectroscopy should be equipped with an environmental control system with temperature, humidity, and CO_2_ control, while keeping cells sterile. Media containing phenol red will contribute to the resulting cell signal with an increase in fluorescent background, especially if an incident light at the near‐infrared region is used for Raman measurement. To avoid such issues, replacing culture media with phenol red‐free media is practiced before Raman experiments. Fixed cells are often immersed in phosphate‐buffered saline (PBS), which does not have a significant influence on the resulting Raman spectra. However, the fixation process is known to alter innate cell signals (Hobro and Smith 2017; Meade et al. 2010). The drying process changes the water content in cells, which contributes to the changes in cell signal (Abazari et al. 2014). For Raman measurement of tissues, various preparation methods are practiced specific to their associated organs, thereby contributing different degrees of changes in the resulting tissue signal. Skin tissues are the most studied parts of the body, especially for aging studies that can be measured directly onto the specimen or from biopsies. However, direct measurement can be challenging because varying melanin contents per individual skin contribute to the Raman signal. Biopsied tissues often undergo freezing followed by cryo‐sectioning for Raman measurement. Depending on organs and protocols, tissues can be embedded in paraffin, polymethylmethacrylate (PMMA), optimal cutting temperature (OCT) compounds, and other materials. For formalin‐fixed, paraffin‐embedded (PFFE) tissues, deparaffinization is often practiced prior to Raman measurement to minimize potential interference of paraffin in tissue signal.

Even following these well‐established protocols, there are limitations to solving problems caused by the biological specimen itself. A laser at a low power should be used to account for damage caused by the irradiated laser, which requires an extended measurement time. This will increase the background noise and decrease accuracy. In addition, biological specimens are not homogenous, thereby the same cells, tissues, or organs will show different spectra at different measurement points, and even at different thicknesses. This issue is even more pronounced for quantitative measurements. To solve this, many repeated measurements are required, but this is not a fundamental solution.

A promising solution to address these problems caused by variations in the specimens is applying artificial intelligence (AI) for the analysis of Raman spectra. Many studies have already been conducted using AI to analyze Raman data (Kothari et al. 2021; Wu et al. 2021; Bellantuono et al. 2023), which can be applied to aging research.

## 5 Concluding Remarks

Raman spectroscopy has demonstrated its applicability in aging research at both cellular and tissue levels. It enables analyzing biochemical changes within tissues and cells, enabling researchers to investigate molecular alterations linked to cellular senescence and tissue aging. This non‐invasive, label‐free technique provides detailed information about the molecular composition and structure of biological samples, enhancing the understanding of the aging process and aiding in the development of potential interventions and therapeutic strategies against aging‐associated diseases.

## Author Contributions

S.P., D.Y., and J.H.K. conducted an extensive literature review and collaboratively prepared the initial draft of the manuscript. S.P., D.Y., and J.H.K. wrote the original draft of the article. S.P., D.Y., and J.H.K. prepared the figures. S.P., D.Y., and J.H.K. reviewed and edited the article.

## Conflicts of Interest

The authors declare no conflicts of interest.

## Data Availability

This review article does not involve the creation or analysis of original data. All referenced data are available in the respective published sources, which are cited throughout the manuscript.
